# A Code of Rules for the Prevention of Infectious and Contagious Diseases in Schools

**Published:** 1886-12

**Authors:** 


*A Code of Rules for the Prevention of Infectious and Contagious Diseases in Schools.* Pp. 31. London: J. & A. CHURCHILL.

This very practical and useful Code has been issued by the Medical Officers of Schools' Association.

In a place like Clifton, where there are so many educational establishments, it seems desirable that there should be some agreement or consensus of opinion among medical men respecting those questions which are constantly cropping up, and upon which we are called upon to advise our patients. Such questions are, When is it safe for a boy or girl to return to school, after having had an attack of infectious disease? or, What period of quarantine is necessary after exposure to contagious fevers? and so on.

This Code of Rules furnishes information on these points of a clear and distinctive kind; and as the Code has been prepared after much care and deliberation, and after obtaining answers to an elaborate series of questions sent to every school of importance in the kingdom, it seems to be an authoritative if not a final statement of the whole question. Possibly in some matters of detail as to the periods of incubation and quarantine there may be some difference of opinion; but at all events, if there be any error in the Code on these points, it seems to be on the right and safe side.

The Code, which is in the form of a small pamphlet, contains five sections:

1. On the General Hygiene of Schools.
2. The School Infirmary: its construction, arrangement, &c.
3. The Medical Examination of Scholars on admission.
4. General Precautions on admission.
5. Measures to be adopted when an Infectious Disease appears in a School.

These are all discussed briefly and concisely; but the points which are especially worthy of the attention of practitioners are:

First. The periods of quarantine necessary after exposure to infection. They are as follows:

| | |
|---|---|
| Diphtheria ... ... ... | 12 days' quarantine. |
| Scarlet Fever ... ... | 14 " " |
| Measles ... ... ... | 16 " " |
| German Measles (Rötheln or Epidemic Roseola) ... | 16 " " |
| Chicken Pox ... ... ... | 18 " " |
| Small Pox ... ... ... | 18 " " |
| Mumps ... ... ... | 24 " " |
| Whooping Cough ... ... | 21 " " |

Secondly. With regard to that important question, When may a pupil who has had an infectious disease go home or rejoin the school? the Code gives the following directions:

"A pupil may go home after scarlet fever in not less than six weeks from the date of the rash *if* desquamation has completely ceased, and there be no appearance of sore throat.

"After measles, in not less than three weeks from the date of the rash *if* all desquamation and cough have ceased.

"After German measles (rötheln or epidemic roseola), in two or three weeks.

"After small pox and chicken pox, when every scab has fallen off.

"After mumps, in four weeks from the commencement *if* all swelling has subsided.

"Whooping Cough.—After six weeks from the outset of the whoop, or earlier if all cough has ceased.

"Diphtheria.—In not less than three weeks when convalescence is completed, there being no sore throat or albuminuria.

"Ringworm.—When the *whole* scalp having been examined, no broken-off stumpy hairs are to be detected."

It will be observed that the Code deals with the ordinary exanthemata mainly, and that nothing is said with regard to enteric or typhus fevers.

The book, however, will be found very useful to parents and school authorities, to medical officers of schools, and to practitioners, as an authoritative guide in questions which are often difficult to decide, and about the details of which there is some divergence of opinion among medical men.

